# Revisiting Cementoblastoma with a Rare Case Presentation

**DOI:** 10.1155/2017/8248691

**Published:** 2017-02-26

**Authors:** Vijayanirmala Subramani, Malathi Narasimhan, Suganya Ramalingam, Soumya Anandan, Subhashini Ranganathan

**Affiliations:** Department of Oral Pathology and Microbiology, Faculty of Dental Sciences, Sri Ramachandra University, Chennai, India

## Abstract

Cementoblastoma is a rare benign odontogenic neoplasm which is characterized by the proliferation of cellular cementum. Diagnosis of cementoblastoma is challenging because of its protracted clinical, radiographic features, and bland histological appearance; most often cementoblastoma is often confused with other cementum and bone originated lesions. The aim of this article is to overview/revisit, approach the diagnosis of cementoblastoma, and also present a unique radiographic appearance of a cementoblastoma lesion associated with an impacted tooth.

## 1. Introduction

The cementoblastoma was first described by Dewey in 1927 as an odontogenic tumor of mesenchymal origin [1]. Cementoblastoma is a true neoplasm of cementum which is also designated as true cementoma. It constitutes less than 1% of the odontogenic tumor with distinctive features, occurring almost always in the posterior mandible region and usually affects young people less than 25 yrs. In most of the cases the tumor tends to be associated with the permanent first molar. Cases have been also reported involvement of deciduous teeth [2]. The histopathological features of cementoblastoma closely resemble osteoblastoma [3].

## 2. Case History

**Figure 1:**
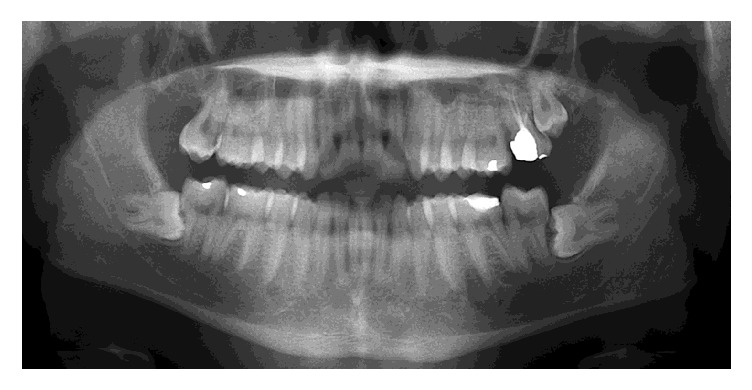
Orthopantomograph shows a well-circumscribed radiolucent mass attached to the lateral root surface of impacted permanent left mandibular first molar.

**Figure 2:**
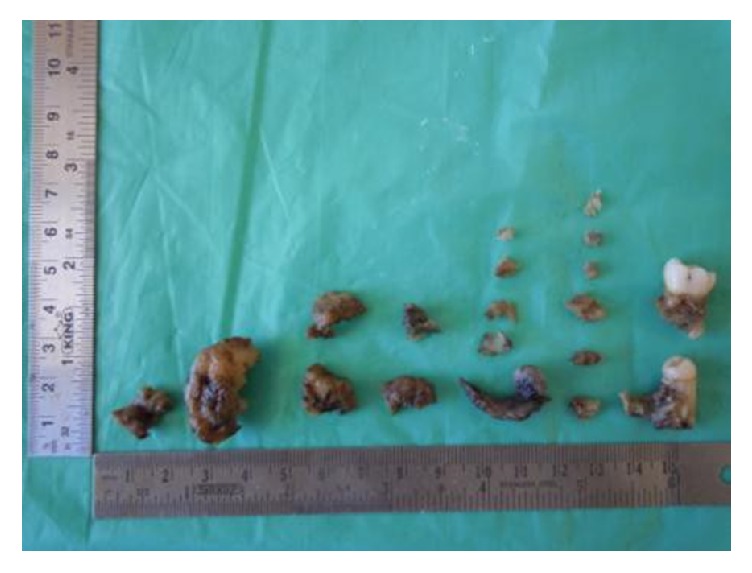
Gross specimen shows multiple bits of hard and soft tissues.

**Figure 3:**
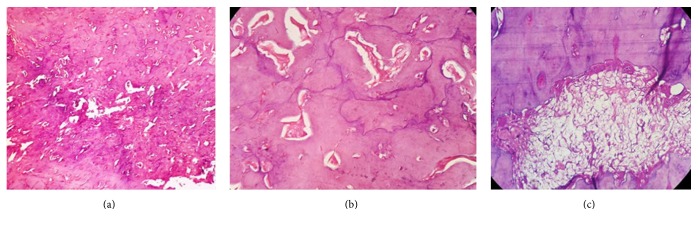
(a) H&E 10x view shows proliferation of cementocytes. (b) H&E 40x view shows basophilic reversal lines. (c) H&E 40x view shows multinucleated resorptive cells in connective tissue stroma.

A 19-year-old male patient complained of swelling in the left body of mandible with an increase in size for the past two months. On clinical examination, an extraoral swelling presents in lower one-third of the face which measures about 3 × 3 cm in size. Intraoral swelling was associated with partially impacted 36. The swelling was well defined and firm-to-hard in consistency, with expansion of lingual and buccal cortex. Tenderness on palpation was noticed. On radiographical examination, OPG revealed a large, well-defined periapical radiolucency arising from the lateral root surface of an impacted permanent left mandibular first molar and second premolar. The lesion was surrounded by a thin, uniform radiopaque line as can be seen in Figure 1. Considering the clinical and radiographical findings, ossifying fibroma was given as the clinical diagnosis. The gross specimen included multiple bits of hard tissues with permanent mandibular first molar and second premolar tooth and 2 bits of soft tissue as can be seen in Figure 2. The largest hard tissue measured approximately 3.5 cm. Hematoxylin and eosin stained sections showed prominent cementoblasts, irregular lacunae, increased active cementoblasts as can be seen in Figure 3(a). The numerous basophilic reversal lines are observed in Paget disease as can be seen in Figure 3(b). Areas of multinucleated giant cells were seen along with areas of loosely arranged vascular connective tissue stroma as can be seen in Figure 3(c). The final histopathological diagnosis was given as cementoblastoma.

## 3. Discussion

Cementoblastomas are slow growing lesions with unlimited growth potential. They are odontogenic tumors and are derived from ectomesenchymal cells of the periodontium including cementoblasts. These tumors are commonly seen in children and young persons; males are more frequently affected than females, with more occurrences in mandible than maxilla. The tumor usually involves an erupted permanent first tooth [2, 4]. Few studies involving documented cases of cementoblastoma associated with impacted teeth have been listed in Table 1. The most common clinical symptom is a painful swelling at the buccal and lingual/palatal aspect of the alveolar ridges; occasionally it may be asymptomatic. The vitality of the involved tooth remains intact. Cortical expansion and facial asymmetry are also common findings. Lower-lip paresthesia or a pathologic fracture of the mandible is rarely reported [5]. The spectrum of radiographic appearance of cementoblastoma depends on its degree of mineralization. Early-stage lesions generally appear more radiolucent and should be differentiated from the periapical inflammatory lesions like focal sclerosing osteotitis and focal osteomyelitis and in mature stage this lesion may be difficult to distinguish from hypercementosis, cementoossifying fibroma, osteoma, benign osteoblastoma, odontomas and calcifying epithelial odontogenic tumors, and so forth. Hypercementosis/cementum hyperplasia is an excessive amount of cellular cementum deposition in nonneoplastic condition. Hypercementosis is clinically asymptomatic in which a uniform width of radiolucent zone without any cortical bone expansion or perforation is evident radiographically. Literature revealed that cementoblastoma possesses a thin radiolucent rim surrounding the radiopaque mass in the mature lesion. As it matures it obliterates the outline of the root on the X-ray where there is always a radiolucent margin surrounding the cementum. Periapical sclerosing osteomyelitis is limited to the periapex of a nonvital tooth and does not show continuous growth. Root resorption, loss of root outline, and obliteration of the periodontal ligament space are common findings [6]. Histopathologically the tumor mass is characterized by the formation of sheets of cementum-like tissue which contains numerous basophilic reversal lines. The reversal lines are similar to those observed in Paget disease. Cells are also seen enclosing the cementum in irregular spaces. Peripherally, there is a broad zone of unmineralized tissue and surrounding connective capsule [7]. The pathogenesis evolves in three stages. Periapical osteolysis is the first stage followed by a cementoblastic stage and then calcification and maturation. Osteocementum-like material is also formed in other lesions such as osteoblastoma, hypercementosis, and chronic focal sclerosing osteitis. Osteosarcoma has been considered as a histopathological differential diagnosis [8]. Slootweg compared cases of cementoblastoma and osteoblastoma and concluded that from a histologic view the two lesions cannot be separated. According to WHO, cementoblastoma having a direct connection with the radicular surface of the tooth is the most significant finding. Hypercementosis is defined as “a nonneoplastic condition in which excessive cementum is deposited in continuation with the normal radicular cementum.” Hypercementosis and cementoblastoma, despite being two distinctive conditions, can pose a diagnostic challenge when presented with atypical manifestation [9]. The treatment of choice is the surgical removal of the lesion along with the affected tooth structures followed by complete curettage. Recurrences are rare, but Brannon et al. [10] stated that “recurrence is more common when curettage is performed without the extraction of the involved tooth or teeth and also expansion of cortical bone/perforation of the cortex are clinical signs of recurrence.”

## 4. Conclusion

The present case is an association with impacted mandibular first molar which has rarest occurrence for cementoblastoma in a 19 yrs male. These lesions are usually slow growing benign neoplasms. These lesions with unusual clinical and radiographic presentation can lead to a misdiagnosis. The clinicians as well as oral pathologists must bear in mind several possible differential diagnoses due to its unspecific nature. Surgical removal is the treatment of choice and postoperative follow-up is highly recommended.

## Figures and Tables

**Table 1:** Literature review of previous report cases of cementoblastoma associated with impacted teeth.

Author	Age/gender	Clinical sign & symptom	Lesion associated with impacted teeth
Piattelli et al.	35 yrs/F	Pain in lower right mandibular posterior region	Associated with impacted 48
Sumer et al.	46 yrs/M	Pain, trismus, & swelling in mandibular left posterior region	Associated with impacted 38
Chauhan	33 yrs/M	Pain & swelling in lower right back region	Associated with impacted 48
Dinakar et al.	41 yrs/M	Pain & swelling in lower right back region	Associated with partially erupted 48
Present case	19 yrs/M	Swelling with mild pain in lower left posterior regions	Associated with partially erupted 36
